# Role of ASLNC168501 in regulating hair follicle stem cell activity via the AR/miR-128-3p/IGF-1 pathway

**DOI:** 10.1186/s13287-026-04905-w

**Published:** 2026-01-27

**Authors:** Xuewen Chen, Jingxiu Chai, Xuan Wang, Leimeng Gan, Qing Zhang, Hao Luo, Ling Wu, Yuchong Chen

**Affiliations:** 1 Department of Dermatologic Surgery, Shanghai Skin Disease Hospital, Tongji University School of Medicine, Shanghai, 200443 China; 2 Department of Hepatobiliary and Pancreatic Surgery, Shuguang Hospital, Shanghai University of Traditional Chinese Medicine, Shanghai, 201203 China

**Keywords:** Androgenetic alopecia (AGA), miR-128-3p, Androgen receptor (AR), IGF-1, Hair follicle stem cells, Dermal papilla cells (DPCs), ASLNC168501

## Abstract

**Background:**

Hair follicle stem cells (HFSCs) in androgenetic alopecia (AGA) patients exhibit functional impairment, reduced quantity, dysregulation, and androgen sensitivity, which hinder therapeutic strategies targeting HFSCs activation for hair regeneration. This study aims to elucidate the molecular mechanisms underlying HFSCs dysfunction in AGA and identify novel therapeutic targets.

**Methods:**

We compared the expression of insulin-like growth factor 1 (IGF-1) in hair follicle tissues between AGA patients and healthy controls, analyzing transcriptional and protein-level differences. Bioinformatics, luciferase assays, and correlation analyses were employed to investigate the AR/miR-128-3p/IGF-1 pathway. Mechanistic studies were conducted using dermal papilla cells (DPCs) from both AGA patients and normal donors, which included RNA interaction assays and functional validation. Furthermore, the mechanism was validated by assessing the phenotypic changes in HFSCs co-cultured experiments. In vivo experiments in AGA mice were performed to evaluate hair follicle regeneration following ASLNC168501 overexpression.

**Results:**

IGF-1 expression was markedly reduced in hair follicles of AGA patients, with transcriptional alterations occurring later than changes at the protein-level alterations. Dysregulation of the AR/miR-128-3p/IGF-1 pathway in DPCs was identified as a key driver of HFSCs dysfunction: AR transcriptionally activates miR-128-3p, which in turn suppresses IGF-1 by binding to its 3’UTR. Consequently, the ability of IGF-1 to sustain and support HFSCs activity is impaired. The endogenous ASLNC168501 functions as a ceRNA, sequestering miR-128-3p and thereby restoring IGF-1 expression and secretion. Exogenous overexpression of ASLNC168501 in DPCs significantly promoted the self-renewal, proliferative and differentiation potential of co-cultured HFSCs in vitro and reversed hair follicle atrophy in AGA mice.

**Conclusions:**

Our findings demonstrate that loss of ASLNC168501 accelerates the progression of AGA by activating AR/miR-128-3p/IGF-1 pathway activation. Acting as a pathway-independent RNA, ASLNC168501 holds a target significant therapeutic potential for restoring HFSCs function and promoting hair follicle regeneration. This finding highlights a novel molecular target and contributes to the advancement of precision medicine strategies for androgen-related alopecia.

**Supplementary Information:**

The online version contains supplementary material available at 10.1186/s13287-026-04905-w.

## Introduction

Dysfunction of HFSCs differentiation is a key contributor to androgenetic alopecia (AGA), a condition characterized by a shortened anagen phase, prolonged telogen phase, and miniaturization of hair follicles. Miniaturization of hair follicles results from disruptions in the growth cycle, primarily due to the accumulation of dihydrotestosterone (DHT). Testosterone and DHT influence hair follicle activity by binding to androgen receptors (AR) in follicular cells. Insulin-like growth factor 1 (IGF-1), predominantly expressed in DPCs, is regulated by androgens [1], promotes the anagen phase [2], and plays a central role in the hair cycle. It regulates follicular proliferation, hair shaft differentiation, and tissue remodeling, while also serving as a crucial mitogenic and morphogenetic regulator in hair follicle biology. As a result, IGF-1 has emerged as a promising therapeutic target for hair loss conditions, including AGA and alopecia areata [3, 4].

Recent studies suggest that androgen-mediated regulation of IGF-1 may involve both transcriptional and post-translational mechanisms [5]. AR can directly regulate miRNAs by binding to androgen response elements (AREs) in the promoters of their target genes, leading to either activation or repression of transcription. For instance, in prostate cancer, AR has been shown to negatively regulate the expression of β-catenin by enhancing miR-4496 expression through direct binding to AREs in the promoter of miR-4496 [6]. In the context of AGA, miR-221 acts as a key target gene of AR, playing a significant role in suppressing proliferation in dermal papilla cells, dermal stem cells, and in DHT-mediated hair growt [7]. Recent research has shown that miR-122-5p carried by ADSC-Exos counteracts the inhibitory effects of DHT on DPCs and hair follicles, stimulating the proliferation and migration of DPCs. However, underlying mechanisms of miR-122-5p require further validation through experimental and clinical investigations [8]. Despite these findings, the involvement of miRNAs in conjunction with AR and IGF-1 in DPCs in regulating the vitality of HFSCs remains insufficiently understood.

Long non-coding RNAs (lncRNAs), which are crucial for stem cell proliferation and differentiation, play vital roles in various diseases, including AGA. Studies show that AGA patients have reduced levels of RP11-818O24.3, which may aid hair follicle recovery by upregulating FGF2 and PI3K-Akt pathways that promote follicle stem cell survival [9]. Additionally, lncRNA5322 promotes hair follicle cell proliferation and differentiation via the miR-21-mediated PI3K-AKT pathway in HFTs [10]. In this study, we identify ASLNC168501 as a new regulatory factor, expressed at lower levels in AGA patient hair follicles. ASLNC168501 can block the AR/miR-128-3p/IGF-1 pathway in DPCs, inhibiting IGF-1 expression and secretion, ultimately lowering IGF-1 levels in the surrounding microenvironment and impairing its support of HFSCs vitality, acting as a competitive endogenous RNA (ceRNA). Unlike previous research, we aim to explore a cross-cellular repair mechanism for AGA-induced follicular miniaturization at the molecular level, offering new insights into ASLNC168501 as a therapeutic target for AGA.

## Materials and methods

### Patient samples

A total of 24 patients with AGA with a Hamilton-Norwood baldness scale score of 3–4 and an age range of 23–49 (median age 33.42), were recruited for this study, along with 24 healthy controls (age range of 23–45, median age of 32.83). Patients diagnosed with systemic diseases were excluded from the study. Scalp hair follicles were obtained from patients undergoing hair transplantation surgery using follicular unit extraction (FUE) techniques. A total of 20–50 hair follicles were collected per participant. Sampling sites were the frontal balding and occipital non-balding scalp in AGA patients, and the frontal non-balding scalp in controls. After the hair follicle tissues were collected, using microsurgical techniques, dermal papillae and follicular matrices were carefully isolated from the hair bulbs for subsequent culture of DPCs and HFSCs, respectively. A portion was directly used for the preparation of DPCs and HFSCs, while the remaining tissues were immediately transferred to liquid nitrogen for storage. Total RNA was subsequently extracted for the quantification of mRNA levels of AR, IGF-1, and ASLNC168501. In parallel, total protein was extracted for the measurement of AR and IGF-1 protein levels. These data will be utilized for the correlation analysis between the expression of ASLNC168501 and IGF-1 protein. All study participants provided written consent, and the study protocol was approved by the research ethics board at Shanghai Skin Disease Hospital, Tongji University, Shanghai, China, in accordance with the principles of the Declaration of Helsinki. This study was approved by the Ethics Committee of Shanghai Skin Disease Hospital, Shanghai, China.

### Cell culture

Human scalp hair follicles were obtained from donors with informed consent and ethical approval. The follicles were washed with sterile PBS to remove blood and debris. Using microsurgical techniques, dermal papillae (DP) and follicular matrices were carefully isolated from the hair bulbs for subsequent culture of DPCs and HFSCs, respectively. For DPCs isolation, DP tissues were enzymatically digested with Type I collagenase (2 mg/mL) (Sigma-Aldrich, MA, USA) at 37 °C for 30 min, followed by gentle trituration and filtration through a 40 μm cell strainer to obtain a single-cell suspension. The suspension was then centrifuged, and the resulting pellet was resuspended in complete medium consisting of DMEM/F12 supplemented with 10% fetal bovine serum (FBS) (Thermo Fisher Scientific, MA, USA), 1% penicillin-streptomycin, and basic fibroblast growth factor (bFGF, 10 ng/mL) (Abcam, Cambridge, UK). The cells were seeded onto culture plates pre-coated with fibronectin (Abcam) and cultured at 37 °C in a humidified atmosphere with 5% CO₂. Medium was changed every 2–3 days, and DPCs were passaged at 70–80% confluence using 0.05% trypsin-EDTA. For HFSCs isolation, hair follicles were digested with Type I collagenase (2.0 mg/mL) and trypsin (0.25%) at 37 °C for 1 h. The resulting cell suspension was filtered through a 40 μm strainer and then labeled with anti-CD34 and anti-CD200 antibodies (Abcam), before being sorted by fluorescence-activated cell sorting (FACS). The purified HFSCs were cultured in DMEM/F12 supplemented with 10 ng/mL epidermal growth factor (Abcam) and 5 µg/mL fibronectin, and maintained at 37 °C in a humidified atmosphere with 5% CO₂. Medium was refreshed every 2–3 days under strict aseptic conditions.

### Reverse transcription quantitative polymerase chain reaction (RT-qPCR)

Total RNA was extracted from the cells and tissues, and reverse transcription was performed using M-MLV reverse transcriptase to synthesize cDNA. RT-qPCR was carried out using the SYBR Green qPCR Master Mix (QIAGEN, Venlo, Netherlands) on the CFX96 Touch Real-Time PCR Detection System (Bio-Rad, CA, USA). The thermal cycling conditions were as follows: initial denaturation at 95°C for 10 seconds, annealing at 60°C for 20 seconds, and extension at 72°C for 20 seconds, repeated for 45 cycles. mRNA expression levels were quantified and normalized to the internal control gene β-actin using the 2^−ΔΔCt^ method. For the analysis of ASLNC168501 expression, U6 snRNA was used as the endogenous control. The primer sequences used for RT-qPCR are as follows: AR, forward 5’-GAAATGGGCCCCTGGATGGATAGC-3’, reverse 5’-TCTGGGGTGGAAAGTAATAGTCAA-3’; IGF-1, forward 5’-AGACAGGGGCTTTTATTTCAACAA-3’, reverse 5’-GCGCAATACATCTCCAGCCTCCTT-3’; β-actin, forward 5’-CAAGGCCAACCGCGAGAAGATGAC-3’, reverse 5’-CCAGAGGCGTACAGGGATAGCAC-3’; ASLNC168501, forward 5’-GTGTGGAGGAGGGGGTTCAT-3’, reverse 5’-CATCCTCCGGTTCTTTGCTCA-3’; U6 snRNA, forward 5’-GTGCTCGCTTCGGCAGCACAT-3’, reverse 5’-AGTGGGGAACCCTTCCATGAGG-3’. All qPCR reactions were performed in strict adherence to the manufacturer’s protocols.

### Enzyme-linked immunosorbent assay (ELISA)

To measure the levels of AR and IGF-1 proteins in hair follicle tissues, total protein was first extracted using M-PER Mammalian Protein Extraction Reagent (Pierce, IL, USA) and homogenized. After homogenization, the samples were centrifuged, and the supernatant was collected. Protein concentration was quantified using the BCA method to ensure equal protein loading for each sample. Next, protein levels of AR and IGF-1 were measured using commercially available ELISA kits (Abcam). Standard solutions and test samples were added to the wells of the ELISA plate, with appropriate blank, positive control, and negative control wells. After incubating at 37 °C for 1 h, the plate was washed three times with wash buffer to remove unbound substances. A secondary HRP-conjugated antibody was then added, followed by a 1-hour incubation, and the plate was washed again. TMB substrate solution was added, and the reaction was allowed to proceed for 20 min, or until a visible color change was observed. The reaction was stopped using stop solution, and absorbance (OD value) was measured at 450 nm using a microplate reader. The concentrations of AR and IGF-1 in the samples were calculated based on the standard curve. Finally, the protein levels of AR and IGF-1 were reported, and statistical analysis was performed to evaluate the expression differences between experimental and control groups under various conditions. Throughout the procedure, strict adherence to the manufacturer’s protocol was followed to ensure the accuracy and reproducibility of the results. Additionally, ELISA was employed to measure the levels of KRT15/19 proteins in HFSCs, using a method similar to that described above. The two ELISA kits used for this analysis were sourced from Abnova and Abcam.

### Western blotting

Total proteins were extracted from cells using the M-PER mammalian protein extraction reagent (Pierce, IL, USA). An equal amount of protein (15 µg) was loaded onto 12% SDS-PAGE gels for electrophoresis, and the separated proteins were transferred to nitrocellulose membranes (Millipore). The membranes were then incubated overnight at 4 °C with primary antibodies targeting human AR (1:400), IGF-1 (1:200), and β-actin (1:1000) (all from Abcam). After primary antibody incubation, the membranes were exposed to a secondary HRP-conjugated anti-rabbit antibody (Abcam, 1:3000) for 1 h at room temperature. Following a series of washes, the protein bands were detected using chemiluminescent substrate (Pierce) and the signals were captured on X-ray films. To ensure proper protein loading and normalization, β-actin was used as the internal control.

### Lentivirus-mediated ASLNC168501 intervention experiment

This experiment consisted of four groups: Control (DPCs with no treatment), Lv-NC group (cells infected with Lv-NC), Lv-ASLNC168501 group (cells infected with Lv-ASLNC168501), and Lv-shRNA-ASLNC168501 group (cells infected with Lv-shRNA-ASLNC168501). DPCs were trypsinized and reseeded into 6-well plates at a density of 1 × 10^5^ cells per well. After overnight incubation, recombinant lentivirus was added at a multiplicity of infection (MOI) of 50. The cells were then cultured under normal conditions for 72 hours. At least three biological replicates were performed for each group to ensure data reliability. The recombinant lentiviruses, Lv-ASLNC168501 and Lv-shRNA-ASLNC168501 (Genomeditech, Shanghai, China), were used to either express or silence ASLNC168501 in the DPCs. Lv-NC served as the control virus, which, after infecting the cells, undergoes transcription and Dicer processing to ultimately generate the mis-translated siRNA-ASLNC168501 sequence (5’-UGCACAUGUGCUGUCAAAU-3’). Seventy-two hours post-infection, GFP expression was observed under a fluorescence inverted microscope to assess the efficiency of lentiviral infection. Subsequently, total RNA was extracted from the cells and used to measure ASLNC168501, AR, and IGF-1 mRNA levels via RT-qPCR. Total protein was also extracted and used for Western blot analysis to assess the expression of AR and IGF-1 proteins.

### Luciferase reporter assays

#### Evaluation of the effect of AR on IGF-1 promoter activity and its impact on protein translation through the 3’ UTR

To begin, we cloned the IGF-1 promoter into a luciferase expression vector to construct the recombinant plasmid pGL3-pro-IGF-1. This vector contains a luciferase gene positioned downstream of the IGF-1 promoter, creating a luciferase-based reporter system. When transfected into mammalian cells, if the IGF-1 promoter is activated, the transcription and protein expression of the luciferase gene are significantly enhanced. Therefore, after co-transfection with pcDNA3.1-AR into DPCs, we can assess whether AR affects the activity of the IGF-1 promoter by measuring changes in luciferase activity within the cells. In parallel, we used human cDNA as a template to PCR-amplify the 3’UTR of human IGF-1 mRNA. This was then cloned downstream of the luciferase gene in a luciferase expression vector to generate the recombinant plasmid pGL3-3’UTR-IGF-1. We co-transfected this plasmid with either pcDNA3.1-AR or siRNA-AR (5’- GAUACUGCUGAGUAUUCCC-3’) into DPCs. Subsequently, we assessed whether AR interacts with the IGF-1 mRNA 3’UTR by evaluating changes in luciferase activity in the cells. One day before transfection, logarithmic-phase DPCs were seeded at a density of 5 × 10⁴ cells per well in a 6-well plate. After overnight incubation under normal conditions, transfection was carried out. The quantities of transfection reagent, DNA, and RNA, as well as the transfection protocol and reagent volumes, were strictly followed according to the Lipofectamine2000 transfection reagent (Thermo Fisher Scientific) manual. After 48 h post-transfection, cells were lysed and luciferase activity was measured using the Dual-Luciferase Reporter Assay System (Promega, Madison, WI, USA).

#### Evaluation of the interaction between miR-128-3p and the IGF-1 3’ UTR

The human IGF-1 3’ UTR, which contains the predicted miR-128-3p binding sites, was amplified from human cDNA. The PCR product was subsequently cloned downstream of the luciferase gene to construct the wild-type reporter plasmid pGL3-wt-3’UTR(IGF-1). To investigate the functional relevance of the miR-128-3p binding sites, mutations were introduced in the miR-128-3p binding sequence (5’-CACUGUG-3’) to 5’-GUGACCU-3’, creating the mutant reporter plasmid pGL3-mt-3’UTR(IGF-1). All RNA oligonucleotides, including the miR-128-3p mimic (5’-UGGCAGUGUCUUUGGUUUGGGUtt-3’), miR-128-3p inhibitor (5’-ACCCAAACCAAAGACACUGCCAtt-3’), and negative control (NC) (5’-UGGCAGUGUCUUUGGUUUGGGUtt-3’), were synthesized by Sangon Biotech (Shanghai, China) with a 3’-terminal “tt” overhang to enhance RNA stability. For the transfection experiment, DPCs were co-transfected with either pGL3-wt-3’UTR(IGF-1) or pGL3-mt-3’UTR(IGF-1) plasmids along with miR-128-3p mimic, inhibitor, or NC using Lipofectamine 2000, following the manufacturer’s instructions. Forty-eight hours post-transfection, the cells were lysed, and luciferase activity was measured to assess the effect of miR-128-3p on the IGF-1 3’ UTR activity.

#### Evaluation of the impact of ASLNC168501 on the interaction between miR-128-3p and IGF-1 3’- UTR

Building on the co-transfection of pGL3-wt-3’UTR(IGF-1) or pGL3-mt-3’UTR(IGF-1) plasmids with miR-128-3p mimic and inhibitor, pcDNA3.1-ASLNC168501 (used for ASLNC168501 overexpression) was also transfected into DPCs. The potential effect of ASLNC168501 on the interaction between miR-128-3p and the IGF-1 3’ UTR was evaluated by comparing changes in luciferase activity in the cells before and after the co-transfection with pcDNA3.1-ASLNC168501.

#### Comprehensive evaluation of AR binding and regulation of miR-128-3p promoter activity

To investigate the interaction between AR and the miR-128-3p promoter, we began by retrieving the genomic location of miR-128-3p from the NCBI database. We selected a 2500-base pair region upstream of the transcription start site as the candidate promoter sequence. Using the “promoter2.0” tool, we predicted the core promoter region, and further employed “JASPAR” to identify potential AR binding sites (AREs) within the miR-128-3p promoter. Subsequently, the miR-128-3p promoter sequence was cloned into a luciferase expression vector to generate the recombinant plasmid pGL3-pro-miR-128-3p. This vector includes the luciferase gene positioned downstream of the miR-128-3p promoter, allowing for the construction of a luciferase-based reporter system. Upon transfection into mammalian cells, activation of the miR-128-3p promoter leads to enhanced transcription and subsequent protein expression of the luciferase gene. To assess the regulatory effect of AR on miR-128-3p promoter activity, we co-transfected DPCs with either pcDNA3.1-AR (for overexpression) or siRNA-AR (for knockdown) and measured the resulting changes in luciferase activity. These experiments enabled us to evaluate the influence of AR on the transcriptional activity of the miR-128-3p promoter.

### RNA Immunoprecipitation (RIP) assay

To evaluate the direct binding of AR and IGF-1 to ASLNC168501, RIP was performed. Hair follicle tissue from AGA patients was lysed in RIP buffer with RNase inhibitors, and the lysate was incubated with an AR/IGF-1-specific antibody (or IgG as a negative control). Protein A/G beads (Thermo Fisher) were used to capture the antibody-protein complex, and after washing, RNA-protein complexes were eluted and extracted. The RNA was reverse transcribed into cDNA and analyzed by quantitative PCR to measure RNA enrichment in the AR/IGF-1 immunoprecipitate. A significant increase in RNA fragments belonging to ASLNC168501 expression in the AR/IGF-1-bound fraction indicates a direct interaction between AR/IGF-1 and the ASLNC168501. The primer sequences used for PCR amplification are as follows: forward primer: 5’-TGCAGAGAACAGACCCCATACT-3’, reverse primer: 5’-GCCCCCACTGTGCTCTCAAT-3’. Additionally, this method was employed to assess whether there is direct binding between the AR and IGF-1 mRNA 3’ UTR regions within the same tissue. The procedure is largely consistent with the one described above, with the main difference being that the primer sequences used for PCR detection in the final step are as follows: forward primer: 5’-CATTTTTGCCCTCTGCCTGTT-3’, reverse primer: 5’- ATCTGAGTCATTCTGCTGTAGTAT-3’. These primers are complementary to the human IGF-1 mRNA 3’ UTR region, and it is assumed that the amplified product will be 466 bp in size.

### CHIP-PCR

This experiment is primarily used to confirm whether there is a direct binding between AR and the miR-128-3p promoter, serving as a necessary complement to bioinformatics analysis and luciferase reporter gene assays. DPCs were crosslinked with 1% formaldehyde for 10 min, quenched with 125 mM glycine, and lysed in ChIP lysis buffer. Chromatin was sheared by sonication to 200–500 bp fragments. Immunoprecipitation was performed using 5 µg AR antibody overnight at 4 °C, followed by incubation with 50 µL protein A/G beads for 1 h. After washing, protein-DNA crosslinks were reversed by incubating at 65 °C for 6 h, and the DNA was purified using a QIAquick PCR purification kit (Qiagen, Hilden, Germany). For PCR, 3 µL of the purified DNA was amplified in a 25 µL reaction containing 12.5 µL 2×PCR Master Mix (Thermo Fisher) and 0.5 µM primers (forward: 5’-TTAGAAGAAGGCTATTGAC-3’, reverse: 5’- GTGGCCATCATGAAAGTGAA-3’). PCR conditions were: 95 °C for 5 min, 35 cycles of 95 °C for 30 s, 58 °C for 30 s, 72 °C for 30 s, and a final extension at 72 °C for 5 min. PCR products were analyzed by 2% agarose gel electrophoresis, with the expected 75 bp product indicating AR binding to the miR-128-3p promoter.

### Impact of ASLNC168501 overexpression on K15 and K19 expression in HFSCs

Primary HFSCs were obtained from both normal individuals and AGA patients and cultured in complete medium for 48 h. Following this, HFSCs were examined under a microscope, and differences in the expression of CD34 and CD200 proteins were analyzed using immunofluorescence staining. Subsequently, a co-culture system was established using a Trans-well plate with a pore size of 0.4 μm (Corning, NY, USA). After 72 h of infection with recombinant viruses Lv-NC or Lv-ASLNC168501, DPCs were seeded into the lower chamber at a density of 2 × 10⁵ cells per well, while HFSCs were seeded into the upper chamber at a density of 1 × 10⁵ cells per well. The co-culture system was maintained under normal conditions. On days 1, 3, 7, and 14 after incubation, HFSCs from the upper chamber were collected, and total proteins were extracted. The levels of K15 and K19 proteins were measured by ELISA. HFSCs collected from the upper chamber after 48 h of co-culture will also be used for proliferation activity and sphere formation assay, as well as for evaluating cellular changes through EdU incorporation and cell cycle analyses.

### Cell viability assessment using CCK-8 assay

HFSCs were trypsinized and seeded into 96-well plates at a density of 5 × 10⁴ cells per well. After overnight incubation, recombinant lentivirus was added at a MOI of 50, and the cells were cultured for 72 h under normal conditions. Cell viability was assessed using the Cell Counting Kit-8 (CCK-8) assay at 24, 48, and 72 h. To each well, 10 µL of CCK-8 reagent was added, and after 2 h of incubation, absorbance was measured at 450 nm using a microplate reader. The optical density (OD) value was directly proportional to cell viability. At least three replicates were performed for each group to ensure statistical reliability.

### Sphere formation assay

The sphere formation assay is a widely used method to assess stem cell self-renewal, proliferative capacity, and pluripotency. The HFSCs were trypsinized with 0.25% trypsin and dispersed into a single-cell suspension, then the suspension (1 × 10⁴ cells/ml) was transferred to non-adherent culture medium and seeded in low-attachment 3 mm culture dishes to encourage the aggregation of cells and the formation of spherical structures under non-adherent conditions. Typically, stem cells aggregate and form regular spherical structures in this environment. The number, size, and morphology of the spheres were observed under a microscope to evaluate the cells’ proliferative and self-renewal abilities.

### Mouse experiment

To assess the effect of ASLNC168501 on hair follicle stem cell function and hair follicle atrophy in AGA, 8-week-old male C57BL/6J mice (purchased from Shanghai SLAC Laboratory Animal Co., Ltd.) were used to establish the AGA model. DHT (1 mg/mouse) was administered subcutaneously once a week for 5 weeks to induce AGA. Concurrently, recombinant lentiviruses (Lv-NC and Lv-shRNA-ASLNC168501) were injected subcutaneously at a dose of 50 µL/mouse (1 × 10^7 ifu/µL), with the same weekly injection frequency as DHT (injected every Thursday). Following the treatment, K17 expression was evaluated using immunofluorescence to assess hair follicle stem cell function. Histological analysis was conducted using HE staining to examine changes in hair follicle morphology and hair density in skin sections. The number of animals in each group was set to 6 (*n* = 6). At 2 weeks after treatment, mice were euthanized by intraperitoneal injection of 1% pentobarbital sodium under excessive anesthesia (100 mg/kg) and their skin was extracted for subsequent experiments. Throughout the study, the mice were housed in a pathogen-free environment with access to food and water, and were maintained on a 12-h light/dark cycle in a temperature-controlled room. The work has been reported in line with the ARRIVE guidelines 2.0.

### Statement

The work has been reported in line with the ARRIVE guidelines 2.0.

### Statistical analysis

The results are presented as the mean ± standard deviation (SD) from three independent experiments. Statistical analysis was performed using SPSS (version 20.0; IBM Corp., NY, USA) and GraphPad Prism 7.0 (GraphPad Software, Inc., CA, USA). Comparisons between groups were made using a two-tailed Student’s t-test or one-way analysis of variance (ANOVA), followed by Tukey’s post-hoc test for pairwise comparisons. A p-value of less than 0.05 was considered to indicate statistical significance.

## Results

### AR and IGF-1 expression in hair follicle tissues exhibits contrasting trends in normal individuals and AGA patients

**Fig. 1:**
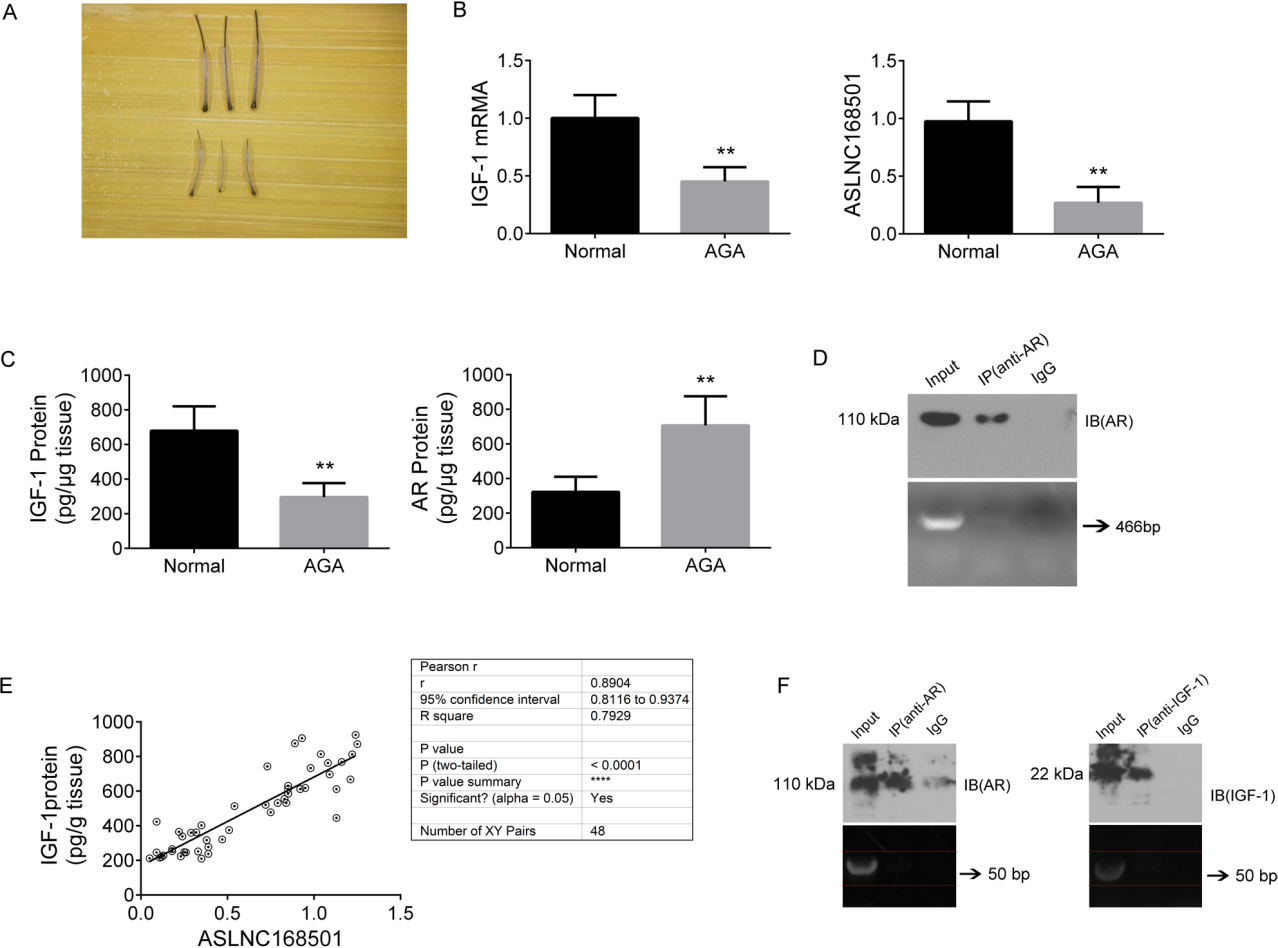
Analysis of IGF-1 and AR Expression in Hair Follicle Tissue from Different Patient Groups and Their Interaction Assays. **A** Sites for Tissue Sample Collection: The top row shows some hair samples from normal individuals, and the bottom row from patients with AGA. **B** RT-qPCR Analysis of Gene Expression. Relative expression levels of IGF-1 mRNA and ASLNC168501 in hair follicle tissues from each group were determined by RT-qPCR. β-actin and U6 served as internal references. Quantification was performed using the 2^−ΔΔCt^ method. **C** ELISA Measurement of IGF-1 and AR Protein Levels. IGF-1 and AR protein levels were measured by ELISA in hair follicle tissues from each group (*n* = 24 per group). **D** RIP assay was performed to confirm the interaction between AR and the 3′UTR of IGF-1 mRNA in tissue samples. A 5 µL PCR product was run on a 2% agarose gel, with the 466 bp band corresponding to the expected target amplified by the primers. **E** Correlation Analysis. Expression correlation between ASLNC168501 and IGF-1 protein levels was analyzed in all hair follicle tissue samples (24 samples from normal individuals and 24 from AGA patients). **F** RIP Assay. RT-PCR products (5 µL) were separated by 2% agarose gel electrophoresis. Data are presented as mean ± SD. Statistical significance was determined as *p* < 0.05 (*) and *p* < 0.01 (**)

RT-qPCR results show that the mRNA level of IGF-1 is significantly lower in the hair follicle tissues of AGA patients compared to normal individuals (*p* < 0.01) (Fig. 1A, B). ELISA results indicate that the IGF-1 protein level is also significantly reduced in the hair follicle tissues of AGA patients compared to normal individuals (*p* < 0.01), while the AR protein level is significantly higher in AGA patients (*p* < 0.01) (Fig. 1C).

### AR inhibits IGF-1 expression post-transcriptionally by interacting with the 3’ UTR of IGF-1 mRNA

Luciferase activity assays show that transfection with pGL3-pro-IGF-1 significantly increases luciferase activity in DPCs (*p* < 0.01 vs. control group). However, transfection with pcDNA3.1-AR does not affect luciferase activity in pGL3-pro-IGF-1-transfected DPCs (*p* > 0.05), indicating that AR does not impact IGF-1 promoter activity (Figure S1A). In contrast, transfection with pGL3-3’UTR-IGF-1 increases luciferase activity in DPCs (*p* < 0.01 vs. control group). Furthermore, transfection of pcDNA3.1-AR or siRNA-AR can respectively inhibit or enhance luciferase activity in pGL3-3’UTR-IGF-1-transfected DPCs (*p* < 0.01 vs. pGL3-3’UTR-IGF-1 group), suggesting that the AR can positively regulate IGF-1 protein expression, and this effect depends on the presence of the 3’ UTR of IGF-1 (Figure S1B). The subsequent RIP-PCR analysis revealed that no fragments corresponding to the IGF-1 3’ UTR were detected in the RNA bound by the AR protein following IP purification (Fig. 1D). In conclusion, the interaction between AR and the IGF-1 mRNA 3’ UTR does not occur directly.

### MiR-128-3p regulates IGF-1 expression by binding to its 3’ UTR

Bioinformatics analysis reveals that the seed region of miR-128-3p has a 7-base binding site in the 3’UTR of IGF-1 mRNA (Figure S1C. Luciferase assays show that miR-128-3p mimics and inhibitors can respectively suppress or enhance luciferase activity in DPCs transfected with pGL3-wt(3’UTR)-IGF-1 (*p* < 0.01, vs. pGL3-wt(3’UTR)-IGF-1 group. In contrast, no significant effect on luciferase activity was observed in cells transfected with pGL3-mt(3’UTR)-IGF-1 (*p* > 0.05, vs. pGL3-mt(3’UTR)-IGF-1 group) (Figure S1D).

### ASLNC168501 exhibits a positive correlation with IGF-1 protein expression in hair follicles of both normal individuals and AGA patients

ASLNC168501 expression is significantly reduced in the hair follicles of AGA patients (*p* < 0.01, vs. Normal). (Fig. 1B). Correlation analysis revealed a positive relationship association between ASLNC168501 and IGF-1 protein expression across all hair follicle tissues (Fig. 1E).

### ASLNC168501 relieves the Inhibition of IGF-1 expression by miR-128-3p

Bioinformatics analysis identified at least three potential miR-128-3p binding sites within the ASLNC168501 sequence, suggesting that ASLNC168501 functions primarily as a miRNA sponge (Figure S1E). Consistently, luciferase reporter assays showed that transfection of pcDNA3.1-ASLNC168501 markedly increased luciferase activity in DPCs co-transfected with miR-128-3p mimics and pGL3-wt(3′UTR)-IGF-1 (*p* < 0.01, vs. miR-128-3p mimics + pGL3-wt(3′UTR)-IGF-1 group). Moreover, pcDNA3.1-ASLNC168501 further enhanced luciferase activity in cells transfected with miR-128-3p inhibitor and pGL3-wt(3′UTR)-IGF-1 (*p* < 0.05, vs. miR-128-3p inhibitor + pGL3-wt(3′UTR)-IGF-1 group) (Figure S1F).

### ASLNC168501 does not directly interact with either AR or IGF-1

The RIP-PCR assay results revealed that RNA fragments of ASLNC168501 were not detected in the eluates of AR and IGF-1 proteins purified by IP from hair follicle tissues of both normal individuals and AGA patients (Fig. 1F). This indicates that neither AR nor IGF-1 directly binds to ASLNC168501, suggesting that their indirect interaction may be mediated by other bioactive molecules.

### ASLNC168501 upregulates IGF-1 protein expression in DPCs independently of AR regulation

**Fig. 2:**
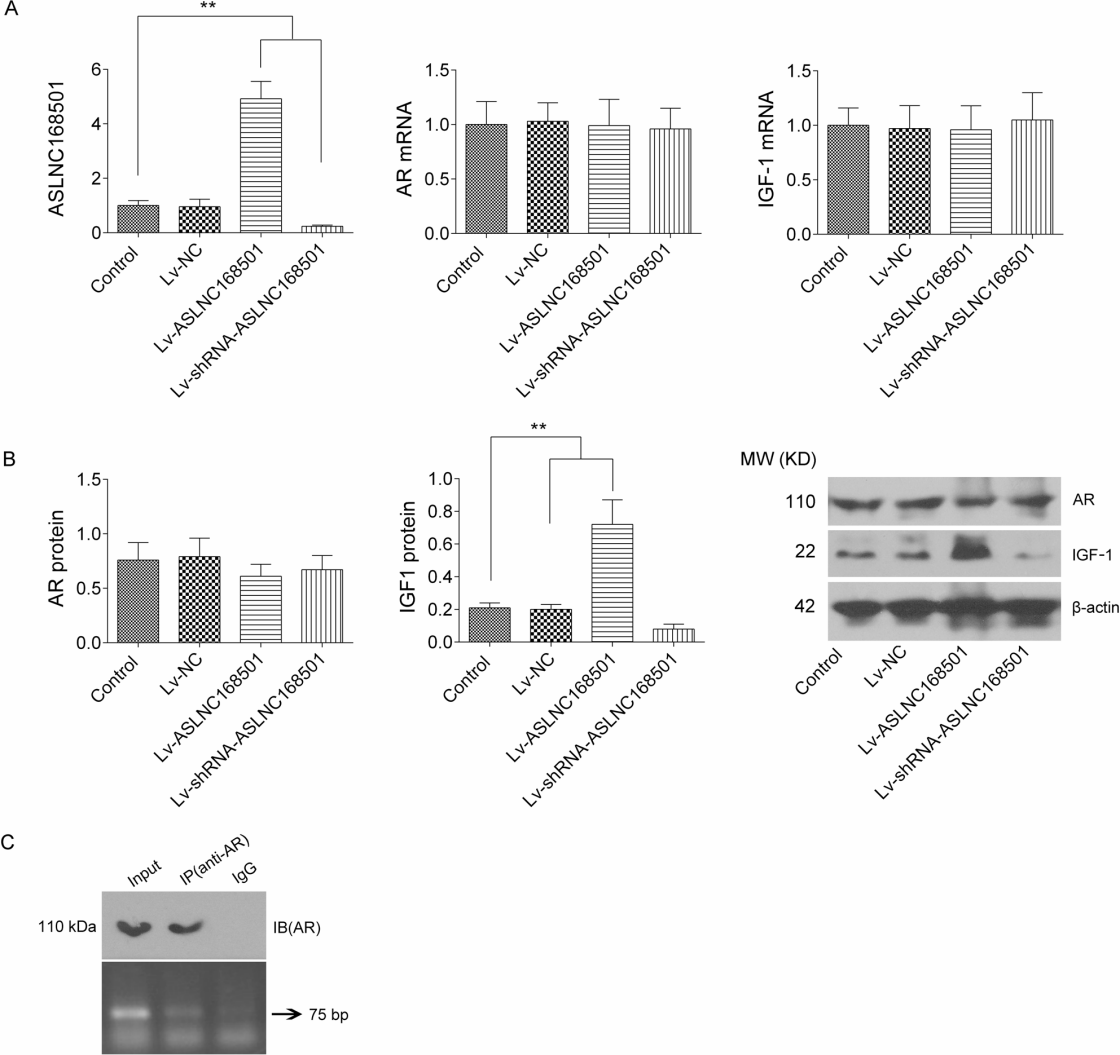
Analysis of the impact of ASLNC168501 expression intervention on the AR/miR-128-3p/IGF-1 pathway in DPCs. **A** RT-qPCR analysis of ASLNC168501, AR, and IGF-1 mRNA levels. U6 or β-actin was used as the internal control. **B** Western blot analysis of AR and IGF-1 protein expression. Total protein was extracted from DPCs 72 h after lentiviral infection, and AR and IGF-1 protein levels were detected by western blotting, with β-actin serving as the loading control. **C** ChIP-PCR was performed to confirm the binding of AR to the miR-128-3p promoter DNA. A 5 µL aliquot of the PCR product was analyzed by 2% agarose gel electrophoresis, with the 75 bp band corresponding to the expected amplicon All experiments were performed with three independent biological replicates (*n* = 3). Data are presented as mean ± SD. Statistical significance: *p < 0.05, **p < 0.01

At 72 hours after lentivirus infection, GFP expression indicated nearly 100% infection efficiency in DPCs. RT-qPCR confirmed significant upregulation of ASLNC168501 in Lv-ASLNC168501-infected cells and downregulation in Lv-shRNA-ASLNC168501-infected cells (*p* < 0.01 vs. control), while AR and IGF-1 mRNA levels remained unchanged (*p* > 0.05, vs. control) (Fig. 2A). Western blotting revealed no significant changes in AR protein levels among groups(*p*>0.05), while IGF-1 protein expression positively correlated with ASLNC168501 levels, being increased in the Lv-ASLNC168501 group and reduced in the Lv-shRNA-ASLNC168501 group (*p* < 0.01, vs. control) (Fig. 2B). Bioinformatics prediction shows that there is an AR binding site (5’-TGTTCT-3’) in the promoter region of miR-128-3p (Figure S1G). Luciferase assay further showed that pGL3-pro-miR-128-3p transfection elevated luciferase activity in DPCs (*p*<0.01, vs.), which was further enhanced by Lv-ASLNC168501 and suppressed by Lv-shRNA-ASLNC168501 (*p* < 0.01 vs. pGL3-pro-miR-128-3p) (Figure S1H). Additionally, ChIP-PCR results showed that DNA fragments from the miR-128-3p promoter region were successfully detected using the DNA template from the IP-purified AR protein eluate (Fig. 2C). This finding corroborates the luciferase assay results, collectively confirming that the transcription factor AR can bind to the miR-128-3p promoter and positively regulate its transcription.

### Overexpression of ASLNC168501 in DPCs can promote the differentiation of co-cultured HFSCs from AGA patients

**Fig. 3:**
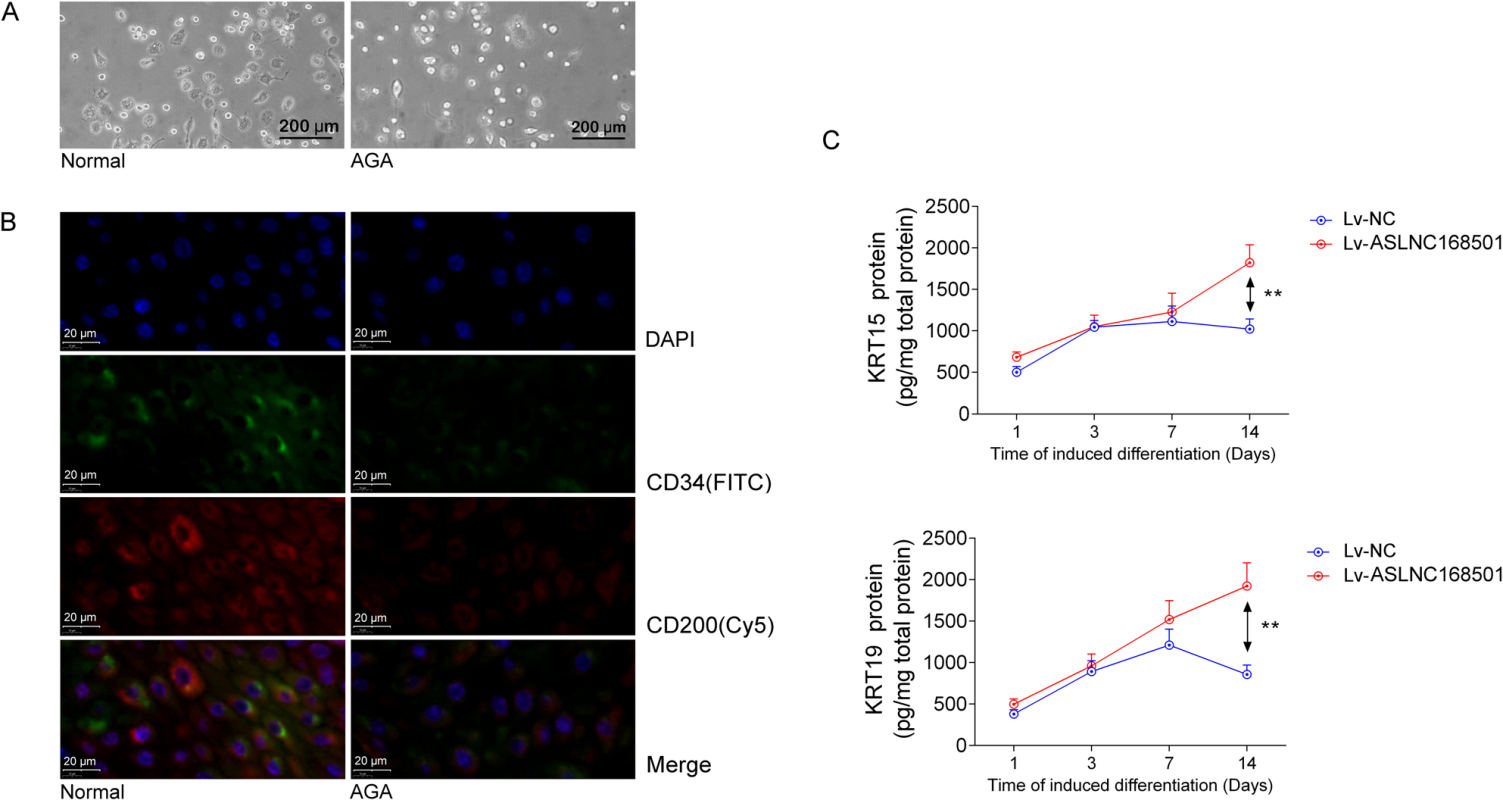
Comparative characterization of HFSCs morphology and stemness markers from distinct sources, and functional assessment of ASLNC168501 in modulating HFSCs differentiation in AGA. **A** Phase-contrast micrographs depicting morphological features of cultured HFSCs. Primary HFSCs were isolated from scalp hair follicles of healthy donors and AGA-affected individuals, then maintained under standard culture conditions for 48 h prior to imaging (scale bar: 200 μm; original magnification: 200×) **B** Immunofluorescence analysis of stem cell surface markers CD34 and CD200. Fixed HFSCs were immunostained with anti-CD200 (red) and anti-CD34 (green) antibodies, with DAPI (blue) counterstaining for nuclear visualization (scale bar: 10 μm; original magnification: 120×). **C** ELISA evaluation of keratinocyte markers K15 and K19 expression profiles. HFSCs derived from AGA patients were transduced with recombinant lentivirus (MOI = 50), followed by protein extraction and immunoblotting. Protein expression levels were quantified by ELISA assay. All experiments were conducted with three biologically independent replicates (*n* = 3). Quantitative data are presented as mean ± SD. Statistical significance was determined by Student’s t-test (*p < 0.05, **p < 0.01)

Microscopic observation of HFSCs from hair follicles of both normal individuals and AGA patients revealed that, compared to normal individuals, the cells from AGA patients were smaller, more rounded or oval in shape, with less cytoplasm and relatively smaller nuclei (Fig. 3A). Immunofluorescence staining results showed that, compared to normal individuals, HFSCs from the AGA group exhibited lower expression levels of HFSCs markers CD34 and CD200 (Fig. 3B). ELISA analysis revealed that the expression of differentiation markers K15 and K19 in co-cultured HFSCs from AGA patients in the upper chamber increased over time (1–14 days) following Lv-ASLNC168501 infection in the lower chamber DPCs. In contrast, in the Lv-NC infection group, the expression of K15 and K19 initially increased but subsequently decreased. At the 14-day time point, both markers exhibited statistically significant differences between the groups (*p* < 0.01) (Fig. 3C). Taken together, these findings demonstrate that overexpression of ASLNC168501 in DPCs alleviates AR-mediated repression of IGF-1 by disrupting the AR/miR-128-3p/IGF-1 axis. The resulting increase in IGF-1 within the co-culture system enhances the activity and differentiation potential of HFSCs, placing the hair follicle in a state more conducive to regeneration and growth. In this context, ASLNC168501 expression in DPCs facilitates IGF-1 upregulation and secretion, thereby maintaining the stemness of neighboring HFSCs and promoting their timely differentiation into functional cells to support hair growth, repair, and regeneration.

### ASLNC168501 promotes hair follicle regeneration in AGA mice

**Fig. 4:**
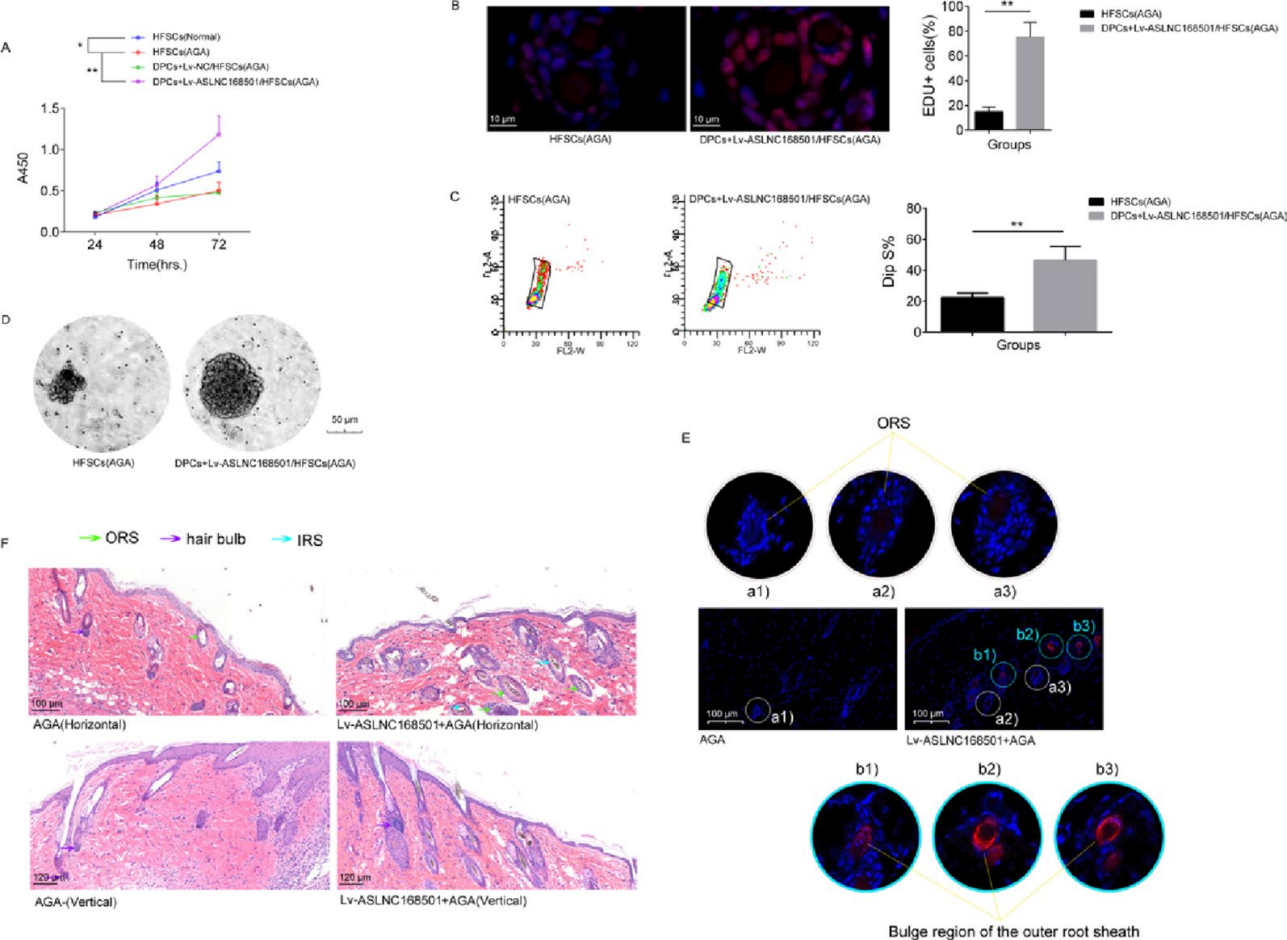
ASLNC168501 enhances HFSCs stemness and promotes hair follicle regeneration in AGA mice. **A** Effects of ASLNC168501 overexpression in DPCs on proliferation of co-cultured HFSCs. Cell viability was assessed by CCK-8 assay following ASLNC168501 overexpression. Data are presented as mean ± SD from three independent experiments (*n* = 3). *p < 0.05, **p < 0.01 vs. control group. **B** EdU incorporation assay. Data are presented as the mean ± SD from three independent experiments (*n* = 3). *p < 0.05, **p < 0.01 vs. the indicated group. **C** Cell cycle analysis. Data are presented as the mean ± SD from three independent experiments (*n* = 3). *p < 0.05, **p < 0.01 vs. the indicated group. **D** Sphere-forming capacity of HFSCs. Representative bright-field images demonstrate the enhanced sphere formation ability of HFSCs co-cultured with ASLNC168501-overexpressing DPCs. Results were consistent across three independent replicates (*n* = 3). **E** K17 expression in regenerated hair follicles. Immunofluorescence staining shows increased K17 protein expression (red) following treatment in hair follicle, with DAPI (blue) nuclear counterstaining (*n* = 6 mice per group). **F** Histological analysis of hair follicle regeneration. H&E staining of dorsal skin sections from AGA mice (*n* = 6 per group) reveals improved hair follicle morphogenesis following treatment

In vitro proliferation assays revealed that HFSCs from AGA patients had significantly lower proliferative activity during the logarithmic phase compared to the normal group (*p* < 0.01). However, compared with the HFSCs (AGA) group, the logarithmic-phase proliferative activity of HFSCs was significantly enhanced in the DPCs + Lv-ASLNC168501/HFSCs (AGA) group (*p* < 0.01, 72 h) (Fig. 4A). Both the EdU incorporation assay and the cell cycle analysis consistently demonstrated that, compared with the HFSCs (AGA) group, the proportion of HFSCs in the S phase was significantly increased in the DPCs + Lv-ASLNC168501/HFSCs (AGA) group (*p* < 0.01) (Fig. 4B, C). Sphere formation assays showed that ASLNC168501 overexpression in DPCs increased the size, shape, and number of spheres formed in co-cultured HFSCs from AGA patients, indicating enhanced self-renewal and proliferative (Fig. 4D). Immunofluorescence and HE staining showed that, compared to AGA mice, Lv-ASLNC168501-infected AGA mice had a significantly higher number of newly formed hair follicles and increased K17 expression (Fig. 4E, F).

## Discussion

In clinical interventions, studies on stem cell-derived components have demonstrated therapeutic efficacy: adipose-derived stem cell (ADSC) extracts significantly improved hair density in AGA patients [11], while mesenchymal stem cell-conditioned medium (MSC-CM) enhanced both hair thickness and density [12]. Our previous research [13] also showed that hair follicle-derived cell transplantation achieved significant repigmentation in vitiligo patients. In contrast, this study focuses on the communication between DPCs and HFSCs. DPCs play a pivotal role in reversing follicular atrophy and restoring hair regeneration in AGA [14]. They modulate hair cycle transitions, particularly the reactivation of the anagen phase, by influencing HFSC phenotypes [15]. Additionally, DPCs secrete key regenerative mediators, such as growth factors, cytokines, and exosomes, which repair the perifollicular microenvironment through immunomodulation and inflammation suppression [11]. Consequently, activating and directing the communication between DPCs and HFSCs in AGA patients can significantly improve the follicular microenvironment and counteract androgen-induced follicle miniaturization. Building on this understanding, our study proposes a novel lncRNA-based strategy (ASLNC168501) to uncover the underlying mechanisms of abnormal protein expression in DPCs of AGA patients, and to explore ways to enhance HFSC self-renewal by reinforcing DPC-HFSC communication.

The IGF-1 are crucial in regulating HFSCs activity. As is well known, AR, particularly through DHT, inhibits anagen and promotes the transition to catagen or telogen, driving AGA development. The expression of AR in DPCs, located at the base of the hair follicle, is associated with the regenerative capacity of the follicle, with AR activation stimulating the proliferation and self-renewal of HFSCs. However, excessive AR activation, particularly via DHT, can lead to hair follicle atrophy and AGA development [16, 17]. In contrast, IGF-1 promotes stem cell proliferation and extends anagen. IGF-1 plays a key role in the growth and differentiation of HFSCs by promoting proliferation, delaying catagen onset, and extending anagen. IGF-1 is predominantly expressed by DPCs and secreted into the microenvironment surrounding HFSCs, its expression is tightly regulated by multiple signaling pathways, including modulation via the AR [1, 3, 4, 7]. Their interaction, especially IGF-1 upregulation, may alleviate androgen-induced follicular damage and support repair, though the precise mechanisms remain unclear. In this study, we identified a novel regulatory pathway involving the AR/miR-128-3p/IGF-1 axis in dermal papilla cells (DPCs). This pathway was activated in both AGA patients and mouse models, leading to a reduction in IGF-1 expression. While these findings align with the traditional theory that suppression of IGF-1 contributes to AGA progression, the more intriguing aspect of our study lies in our discovery that DHT-induced AR activation does not necessarily result in decreased IGF-1 expression. This is because the regulation of the miR-128-3p/IGF-1 axis is influenced by the expression of the epigenetic-associated molecule ASLNC168501. In other words, in individuals with high ASLNC168501 expression, DHT-induced AR activation is less likely to contribute to the progression of AGA.

Our study reveals that in the hair follicle tissue of AGA patients, the nuclear transcription factor AR is activated, and through the positive regulation of miR-128-3p transcription, it inhibits the expression of IGF-1. This disruption results in the loss of stemness in HFSCs, compromising their ability to support the repair of atrophic hair follicles. Notably, we found that the AR/miR-128-3p/IGF-1 pathway is activated in the hair follicles of AGA patients, whereas it is tightly regulated in normal individuals. By constructing an interaction model of pathway components, we identified a lncRNA, ASLNC168501, which is expressed at significantly lower levels in the hair follicle tissue of AGA patients compared to normal individuals. Mechanistic studies revealed that ASLNC168501 acts as a ceRNA, competitively binding to miR-128-3p in the 3’ UTR of IGF-1 mRNA, thereby preventing AR from negatively regulating IGF-1 expression at the transcriptional level. This mechanism is consistent with clinical sample data, which show that AR expression is higher, and IGF-1 expression is lower, in the hair follicle tissue of AGA patients compared to normal controls.

Following a stepwise, evidence-driven workflow from observation to mechanism and validation, we first observed that AR in DPCs reprogrammed transcription, suppressed pro-anagen signaling, and was associated with reduced HFSCs activity. In clinical AGA follicles, AR levels were increased whereas IGF-1 levels were decreased, yielding a consistent inverse correlation relative to controls. Because AR can act as both an activator and a repressor, we tested whether AR directly represses IGF-1; bioinformatic prediction and ChIP–PCR detected no AR occupancy at the IGF-1 promoter, and luciferase assays likewise showed no promoter effect but a robust 3′UTR-dependent effect (Figure. 1D-F), implicating an intermediary microRNA that (i) is transcriptionally regulated by AR and (ii) targets IGF-1. Integrated bioinformatic and experimental analyses identified miR-128-3p as fulfilling these criteria: AR bound and activated the miR-128-3p promoter, and miR-128-3p directly engaged the IGF-1 3′UTR to repress translation (Figure S1A, B,D), thereby defining an AR/miR-128-3p/IGF-1 axis. Across cellular contexts, AR overexpression markedly increased miR-128-3p and reduced IGF-1 in AGA-derived DPCs but had minimal effects in normal DPCs, predicting an additional modulator that is reduced in AGA follicles and interacts with miR-128-3p to constrain the axis, properties consistent with a lncRNA. Differential expression and structural screening nominated ASLNC168501, and subsequent experiments verified that ASLNC168501 is downregulated in AGA follicles and binds miR-128-3p in a ceRNA dependent manner, thereby modulating the AR/miR-128-3p/IGF-1 pathway; overexpression of ASLNC168501 in DPCs restored IGF-1 expression and secretion and increased HFSCs proliferation and differentiation metrics. LncRNAs play a key role in regulating gene expression. To date, multiple mechanisms of cis-regulation have been proposed, including lncRNA molecules scaffolding the recruitment of transcription factors, co-factors, and chromatin-modifying complexes, as well as the act of lncRNA transcription reconfiguring the local three-dimensional chromatin architecture. where they act locally to modulate the expression of neighboring protein-coding genes but lack protein-coding potential [18]. Previous studies have highlighted their involvement in the pathological processes of various diseases, including cancer, immune system-related disorders, and proliferative skin diseases. Linlin Bao demonstrated that RP11-818O24.3 promotes hair follicle recovery and proliferation in an AGA mouse model [9]. Jiachao Xiong showed that overexpression of AC010789.1 enhances the biological function of HFSCs by downregulating miR-21-5p and TGF-β1 expression, while simultaneously upregulating the Wnt/β-catenin signaling pathway [19]. In our study, we observed that the expression of ASLNC168501 and IGF-1 in the hair follicle tissue of both AGA patients and normal individuals remained strongly positively correlated.

Background research indicates that ASLNC168501 is located on chromosome 5 of homo sapiens, according to the GRCh38.p14 Primary Assembly. To date, no studies have been reported regarding its role in biological regulation. Our in vitro experiments revealed that overexpressing or silencing ASLNC168501 could effectively upregulate or downregulate IGF-1 protein expression in DPCs (without significantly affecting mRNA levels), while AR expression showed no notable changes, either at the mRNA or protein level. This result underscores the upstream and downstream relationship between AR and IGF-1. RIP assays indicated that ASLNC168501 does not bind to AR protein nor directly interact with IGF-1 protein. Additionally, luciferase assays confirmed that ASLNC168501 effectively inhibits the binding of miR-128-3p to the IGF-1 3’UTR region, consistent with bioinformatics analysis suggesting ASLNC168501 acts as a miRNA sponge. Collectively, these findings suggest that ASLNC168501 may function as an independent regulator of the AR/miR-128-3p/IGF-1 pathway, highlighting its potential as a therapeutic target for AGA.

Subsequent in vitro functional analyses confirmed that overexpression of ASLNC168501 in DPCs effectively enhances the expression of total K15 and K19 proteins in co-cultured HFSCs from AGA patients, which are commonly regarded as markers of HFSC stemness [20]. We also observed a significant increase in the proliferation activity and their self-renewal capability in the these HFSCs. Moreover, in vivo assessment in AGA mice revealed compelling therapeutic outcomes: ASLNC168501 treatment increased hair follicle density and diameter concomitant with robustly elevated K17 (keratin 17) protein expression in bulge region of outer root sheath of hair follicle, which is a key marker of HFSCs, and strongly associated with the proliferative and regenerative capacities of HFSCs [21, 22]. These findings collectively position ASLNC168501 as a novel regulator of HFSCs homeostasis in androgen-related alopecia.

This study highlights ASLNC168501 as a potential therapeutic target for AGA, while its downregulation in hair follicles also suggests its potential as an early biomarker for disease screening. Future research should address these expression differences from an epigenetic perspective and improve methods to target ASLNC168501 to HFSCs for more effective therapy. Several nanomaterials [23, 24], including gold, silver, PLGA, PEI, liposomes, and carbon nanotubes, can be used for targeted delivery of ASLNC168501 to DPCs and hair follicle. These materials allow precise gene delivery and promote follicle regeneration by enhancing targeting and minimizing side effects. Combining nanomaterials with RNA delivery methods holds great potential for treating AGA and advancing personalized medicine in hair regeneration.

Emerging evidence suggests that decreased expression of ASLNC168501 is a promising biomarker for early AGA screening. LncRNAs, with strong tissue specificity, regulate key hair follicle pathways like Wnt/β-catenin and IGF-1. For example, lysosome-associated lncRNA-LCDR in lung cancer affects lysosomal integrity, potentially contributing to hair follicle miniaturization in AGA [25]. LncRNAs regulate gene expression through chromatin remodeling and miRNA sponging, and their role in monitoring AR-related epigenetic changes in AGA indicates their potential as early biomarkers [26]. The stability of circulating lncRNAs in biofluids enhances their clinical value. Quantitative PCR of lncRNAs in peripheral blood has surpassed traditional methods in tuberculosis diagnosis, and similar profiling of serum or hair follicle fluid could enable non-invasive AGA screening, reducing the need for invasive biopsies [27, 28]. However, challenges remain in standardizing detection methods and validating their roles in AGA. Future studies should integrate multi-omics analyses and AI-driven models to establish lncRNA-based diagnostic frameworks and advance personalized AGA therapies.

## Conclusion

Our study underscores the role of the AR/miR-128-3p/IGF-1 pathway in DPCs under AGA conditions, where its activation accelerates HFSCs stemness loss and follicular atrophy. Our study identifies the lncRNA ASLNC168501 as a pathway-independent ceRNA that mitigates miR-128-3p-mediated IGF-1 suppression, promoting HFSCs proliferation and self-renewal. These findings position ASLNC168501as a potential therapeutic target for regenerating atrophic follicles and an early biomarker for predicting AGA progression.

## Supplementary Information

Below is the link to the electronic supplementary material.

Supplementary Material 1. Figure S1. Bioinformatics analyses and luciferase reporter assays A, B) The interaction between the nuclear transcription factor AR and the promoter or 3′UTR of the IGF-1 gene was verified using luciferase reporter assays. DPCs were co-transfected with reporter constructs and siRNA, and harvested after 48 hours for luciferase activity measurement. C, D) Prediction of miR-128-3p binding sites in the 3′UTR of IGF-1 mRNA, which were further verified by luciferase reporter assays. E, F) Bioinformatics Prediction of miR-128-3p Binding Sites in ASLNC168501, which were further verified by luciferase reporter assays. G, H) A schematic diagram of the Androgen Response Element (ARE) in the miR-128-3p promoter region was presented, followed by validation through luciferase reporter assays. Luciferase activity was measured 48 hours after co-transfection of plasmid DNA and siRNA into DPCs. All experiments were performed with three independent biological replicates (n=3). Data are presented as mean ± SD. Statistical significance: *p < 0.05, **p < 0.01.

## Data Availability

Not applicable.

## Materials availability

This study did not generate new unique reagents.
