# Supplementary material for: Role of ASLNC168501 in regulating hair follicle stem cell activity via the AR/miR-128-3p/IGF-1 pathway
Source: Stem Cell Res Ther. 2026 Jan 27;17:89. doi: 10.1186/s13287-026-04905-w (PMC12918351; doi:10.1186/s13287-026-04905-w)
Supplement: Supplementary file 1 — Supplementary Material 1. Figure S1. Bioinformatics analyses and luciferase reporter assays A, B) The interaction between the nuclear transcription factor AR and the promoter or 3′UTR of the IGF-1 gene was verified using luciferase reporter assays. DPCs were co-transfected with reporter constructs and siRNA, and harvested after 48 hours for luciferase activity measurement. C, D) Prediction of miR-128-3p binding sites in the 3′UTR of IGF-1 mRNA, which were further verified by luciferase reporter assays. E, F) Bioinformatics Prediction of miR-128-3p Binding Sites in ASLNC168501, which were further verified by luciferase reporter assays. G, H) A schematic diagram of the Androgen Response Element (ARE) in the miR-128-3p promoter region was presented, followed by validation through luciferase reporter assays. Luciferase activity was measured 48 hours after co-transfection of plasmid DNA and siRNA into DPCs. All experiments were performed with three independent biological replicates (n=3). Data are presented as mean ± SD. Statistical significance: *p < 0.05, **p < 0.01. [file 13287_2026_4905_MOESM1_ESM.docx]

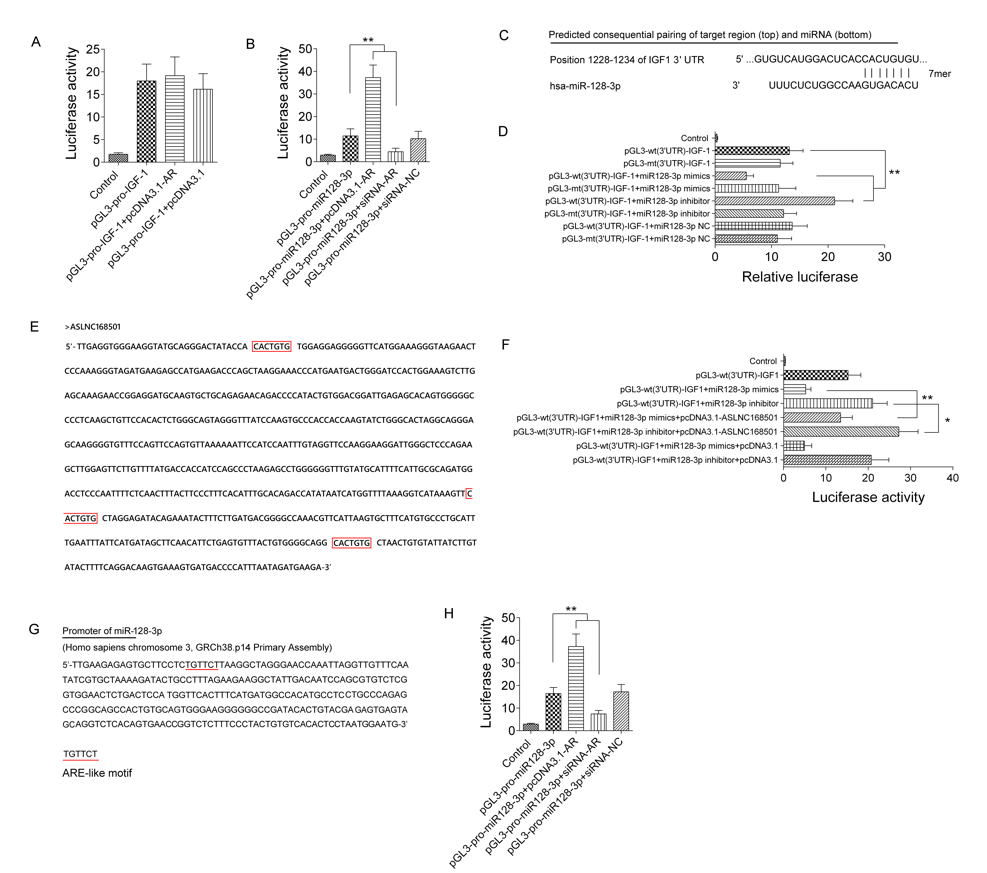


**Figure S1. Bioinformatics analyses and luciferase reporter assays** A, B) The interaction between the nuclear transcription factor AR and the promoter or 3′UTR of the IGF-1 gene was verified using luciferase reporter assays. DPCs were co-transfected with reporter constructs and siRNA, and harvested after 48 hours for luciferase activity measurement. C, D) Prediction of miR-128-3p binding sites in the 3′UTR of IGF-1 mRNA, which were further verified by luciferase reporter assays. E, F) Bioinformatics Prediction of miR-128-3p Binding Sites in ASLNC168501, which were further verified by luciferase reporter assays. G, H) A schematic diagram of the Androgen Response Element (ARE) in the miR-128-3p promoter region was presented, followed by validation through luciferase reporter assays. Luciferase activity was measured 48 hours after co-transfection of plasmid DNA and siRNA into DPCs. All experiments were performed with three independent biological replicates (n=3). Data are presented as mean ± SD. Statistical significance: **p* < 0.05, ***p* < 0.01.
